# Crystal structure and Hirshfeld surface analysis of 2-{[(*E*)-(3-cyclo­butyl-1*H*-1,2,4-triazol-5-yl)imino]­meth­yl}phenol

**DOI:** 10.1107/S2056989021011658

**Published:** 2021-11-09

**Authors:** Mustafa Kemal Gumus, Fatih Sen, Sevgi Kansiz, Necmi Dege, Eiad Saif

**Affiliations:** a Artvin Coruh University, Science-Technology Research and Application Center, 08000, Artvin, Turkey; b Yozgat Bozok University, Sorgun Vocational School, 66100, Yozgat, Turkey; c Samsun University, Faculty of Engineering, Department of Fundamental Sciences, 55420, Samsun, Turkey; d Ondokuz Mayıs University, Faculty of Arts and Sciences, Department of Physics, 55139, Samsun, Turkey; e Department of Computer and Electronic Engineering Technology, Sanaa Community College, Sanaa, Yemen; f Department of Electrical and Electronic Engineering, Faculty of Engineering, Ondokuz Mayıs University, 55139, Samsun, Turkey

**Keywords:** crystal structure, cyclo­but­yl, triazole, salicyl­aldehyde, microwave-assisted synthesis, Hirshfeld surface

## Abstract

The title compound, C_13_H_14_N_4_O, was developed using the reaction of salicyl­aldehyde and 3-amino-5-cyclo­butyl-1,2,4-triazole in ethanol under microwave irradiation. This eco-friendly microwave-promoted method proved to be efficient in the synthesis of 2-{[(*E*)-(3-cyclo­butyl-1*H*-1,2,4-triazol-5-yl)imino]­meth­yl}phenol in good yields and purity. The title compound is a Schiff base that exists in the phenol–imine tautomeric form and adopts an *E* configuration. The three independent mol­ecules in the asymmetric unit (*A*, *B* and *C*) are not planar, the cyclo­butyl and the phenol-imine rings are twisted to each other making a dihedral angle of 67.8 (4)° in mol­ecule *A*, 69.1 (2)° in mol­ecule *B* and 89.1 (2)° in mol­ecule *C*. In each mol­ecule an intra­molecular O—H⋯N hydrogen bond is present, forming an *S*(6) ring motif. A Hirshfeld surface analysis was performed to investigate the contributions of the different inter­molecular contacts within the supra­molecular structure. The major inter­actions are H⋯H (53%), C⋯H (19%) and N⋯H (17%) for mol­ecule *A*, H⋯H (50%), N⋯H (20%) and C⋯H (20%) for mol­ecule *B* and H⋯H (57%), C⋯H (14%) and N⋯H (13%) for mol­ecule *C*.

## Abstract 2

2-{[(*E*)-(3-Cyclo­butyl-1*H*-1,2,4-triazol-5-yl)imino]­meth­yl}phenol was synthesized by an eco-friendly microwave-assisted method that is highly selective and efficient. In the crystal, mol­ecules are linked by N—H⋯N and C—H⋯O hydrogen bonds.

## Chemical context

Imines (Schiff bases) have been extensively used as analytical and medicinal materials (Bülbül *et al.*, 2017 ▸; Singh, 2021 ▸). 1,2,4-Triazoles possess a number of medicinal attributes (Aggarwal & Sumran, 2020 ▸). Taking into account the above considerations, it was decided to merge the chemistry of both parts by reacting 3-amino-5-cyclo­butyl-1,2,4-triazole with salicyl­aldehyde to develop an efficient green protocol for the synthesis of 2-[(*E*)-(5-cyclo­butyl-2*H*-1,2,4-triazol-3-yl­imino)­meth­yl]phenol. In this work, an eco-friendly protocol for the synthesis of Schiff bases from 3-amino-5-cyclo­butyl-1,2,4-triazole and salicyl­aldehyde in ethanol under microwave irradiation was developed. In addition, 2-[(*E*)-(5-cyclo­butyl-2*H*-1,2,4-triazol-3-yl­imino)­meth­yl]phenol was characterized by single crystal X-ray diffraction and investigated using Hirshfeld surface analysis. 
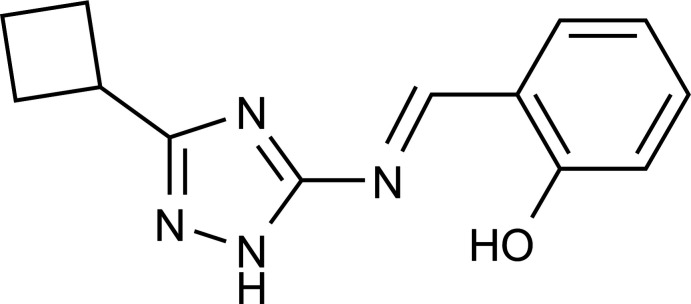


## Structural commentary

**Figure 1:**
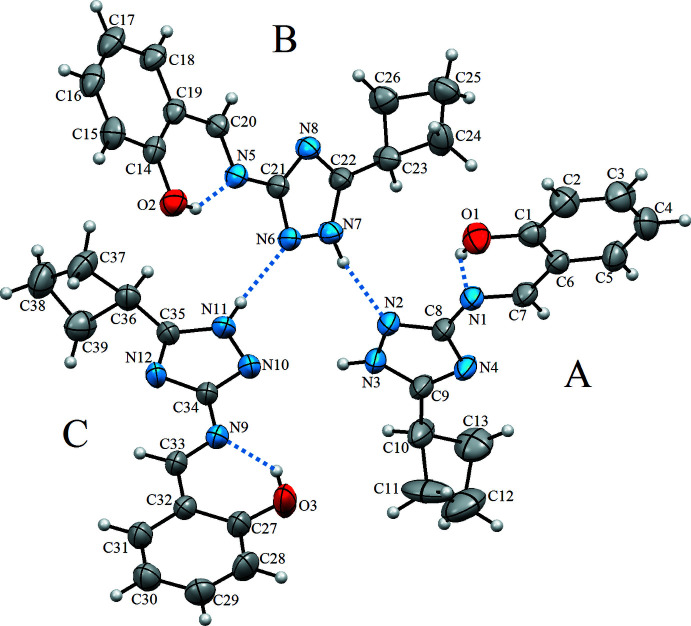
The mol­ecular structure of (I) with the atom labelling. Displacement ellipsoids are drawn at the 30% probability level.

The mol­ecular structure of the title compound, (I), with the atomic numbering scheme is shown in Fig. 1 ▸. The asymmetric unit contains three non-planar mol­ecules. The 1,2,4-triazole and phenol-imine rings are twisted with respect to each other, making a dihedral angle of 18.1 (3)° for mol­ecule *A*. The cyclo­butyl ring is twisted by 73.9 (3) and 67.8 (4)°, with respect to the 1,2,4-triazole, and phenol-imine rings in mol­ecule *A*. The corresponding angles in mol­ecule *B* are 18.7 (3), 74.6 (2) and 69.1 (2)° and 3.2 (4), 85.9 (2) and 89.1 (2)° for mol­ecule *C*. When these angles for the three mol­ecules are compared, it is observed that there is a harmony between them, as well as significant differences, especially in the angles between the phenol-imine and 1,2,4-triazole rings [3.2 (4)° in mol­ecule *C* but around 18° in *A* and *B*] and the phenol-imine cyclo­butyl rings [67.8 (4)° in *A*, 69.1 (2)° in *B* and 89.1 (2)° in *C*]. In the mol­ecules, the C=N group has a strong electron-withdrawing character, as revealed by the double-bond character of the C=N bond [1.276 (6)–1.287 (6) Å] and the single-bond character of C—O [1.349 (5)–1.355 (6) Å] in the phenol–imine tautomer. Furthermore, the azomethine C=N double bond has an *E* configuration. These values and other bond lengths and angles (Table 1 ▸) are in good agreement with those previously reported for C=N and O—C bonds (Bülbül *et al.*, 2019 ▸; Demircioğlu *et al.*, 2019 ▸). The average triazole N—N bond length is 1.353 Å. This length is quite close to the corresponding values reported by Al-Karawi and co-workers [1.343 (4) and 1.353 (6) Å; Al-Karawi *et al.*, 2021*a* ▸,*b* ▸]. In each mol­ecule, the hydroxyl H atom is involved in a strong intra­molecular O—H⋯N hydrogen bond (O1—H1⋯N1, O2—H2⋯N5 and O3—H3⋯N9; Table 2 ▸) forming an *S*(6) ring motif.

## Supra­molecular features

**Figure 2:**
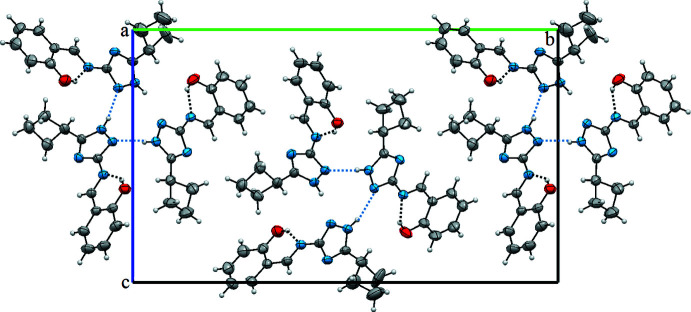
A partial view of the crystal packing of (I) along the *a* axis.

In the crystal, inter­molecular hydrogen bonds N3—H3*D*⋯N10^ii^, N7—H7*D*⋯N2, N11—H11⋯N6 and C10—H10⋯O3^i^ (symmetry codes as in Table 2 ▸) link the mol­ecules into [100] chains. A view of the crystal packing of the structure is shown in Fig. 2 ▸.

## Database survey

There are no direct precedents for the structure of (I) in the crystallographic literature (CSD Version 5.42, update of May 2021; Groom *et al.*, 2016 ▸). However, several related com­pounds have been reported that include (*E*)-*N*-benzyl­idene-1*H*-1,2,4-triazol-5-amine as the main skeleton, *viz*. 5-meth­yl-2-[(1*H*-1,2,4-triazol-3-yl­imino)­meth­yl]phenol (PEVXAS; Brink *et al.*, 2018 ▸), 1-(4-bromo­phen­yl)-*N*-(1*H*-1,2,4-triazol-3-yl)meth­animine (TIVDUA; Kołodziej *et al.*, 2019 ▸), 5-bromo-2-{[(1*H*-1,2,4-triazol-3-yl)imino]­meth­yl}phenol (TIVFAI; Kolodziej *et al.*, 2019 ▸), 4-bromo-2-[(1*H*-1,2,4-triazol-3-yl­imino)­meth­yl]phenol (UZOKIE; Chohan & Hanif, 2011 ▸) and 3,5-bis­(salicyl­idene­amino)-1*H*-1,2,4-triazole methanol solvate (WEFTUX; Cheng *et al.*, 2006 ▸). In addition, 1-[(1*H*-1,2,4-triazol-3-yl­imino)­meth­yl]-2-naphthol (GILYUX; Jia *et al.*, 2013 ▸), which contains a naphthalene fragment instead of benzene, has been reported. In UZOKIE, the hydroxyl-C2 group makes a dihedral angle of 4.48 (3)° with the plane of the 1,2,4-triazole ring system. In addition, there are intra­molecular O—H⋯N contacts in the mol­ecule. Similarly, in WEFTUX, the hydroxyl H atom is involved in an intra­molecular O—H⋯N hydrogen bond, forming an *S*(6) ring motif as in the title compound. The two benzene rings (1 and 3) and the triazole ring (2) in WEFTUX, are almost in the same plane, the angles between rings 1 and 2, and between rings 2 and 3 being 3.7 (2) and 3.3 (2)°, respectively. This latter angle is 4.58 (8)° in PEVXAS. In the structures mentioned above, the twist angles between triazole and phenyl rings are quite small, as in mol­ecule *C* of (I) [3.2 (4)°]; however, for mol­ecules *A* and *B* of the title compound, these angles are over 18°. All compounds were isolated as the phenol-imine (O—H⋯N) tautomeric form, as in (I). The bond lengths of the triazole ring in the studied compound are very similar to those in the other 1*H*-1,2,4-triazole derivatives mentioned above.

## Hirshfeld surface analysis

**Figure 3:**
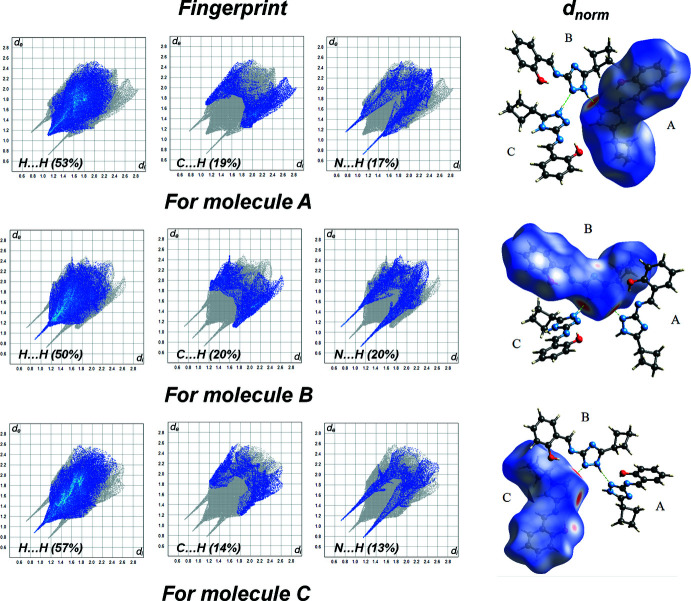
The Hirshfeld surfaces of mol­ecules *A*, *B* and *C* mapped with *d_norm_* showing the N—H⋯N hydrogen-bonded contacts.

**Figure 4:**
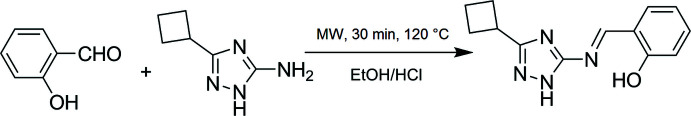
The synthesis of 2-{[(*E*)-(3-cyclo­butyl-1*H*-1,2,4-triazol-5-yl)imino]­meth­yl}phenol, (I).

We performed a Hirshfeld surface analysis and generated the associated two-dimensional fingerprint plots (Spackman & Jayatilaka, 2009 ▸) with *CrystalExplorer17* (Turner *et al.*, 2017 ▸). Hirshfeld surface (HS) analysis is a valuable tool for assessing the strength of inter­molecular inter­actions and for predicting the properties of a crystal and its potential applications (Demir Kanmazalp *et al.*, 2019 ▸; Al-Resayes *et al.*, 2020 ▸). The Hirshfeld surfaces were generated using a standard (high) surface resolution with the three-dimensional *d_norm_* surface mapped over fixed colour scales of −0.6059 (red) to 1.5176 Å (blue) (mol­ecule *A*), −0.6084 (red) to 1.2881 Å (blue) (mol­ecule *B*) and −0.6060 (red) to 1.5351 Å (blue) (mol­ecule *C*), respectively. In Fig. 3 ▸, the red circle on the *d_norm_* surface of mol­ecules *A*, *B* and *C* represents the N—H⋯N inter­actions. The major inter­actions of the compound (Fig. 4 ▸) are H⋯H (53%), C⋯H (19%) and N⋯H (17%) for mol­ecule *A*, H⋯H (50%), N⋯H (20%) and C⋯H (20%) for mol­ecule *B* and H⋯H (57%), C⋯H (14%) and N⋯H (13%) for mol­ecule *C*. It was found that the structure is stabilized by hydrogen bonds (N—H⋯N, O—H⋯N and C—H⋯O).

## Synthesis and crystallization

Salicyl­aldehyde (1.0 mmol), 3-amino-5-cyclo­butyl-1,2,4-triazole (1.0 mmol) and absolute EtOH (2.0 ml) were mixed in a microwave process vial (10 ml), then a 4 *N* solution of HCl in dioxane (one drop) was added. The mixtures were irradiated at 393 K for 30 min. The precipitated solid was filtered, washed with cold ethanol and dried at 353 K. The title compound was obtained in the form of a pale-yellow solid in 92% yield. It was recrystallized from ethanol (m.p. 448–449 K). The reaction scheme is shown in Fig. 4 ▸. The microwave experiment was carried out using a monomode Anton Paar Monowave 300 microwave reactor (2.45 GHz) in a G10 sealed microwave process vial (10 ml). The reaction temperatures were monitored by an IR sensor. After completion of the reaction, the vial was cooled to 323 K by air jet cooling.

IR (Shimadzu Prestige–21 Fourier spectrometer, ATR, cm^−1^): 759, 991, 1030, 1076, 1276, 1562, 1612, 2986, 3040.

^1^H NMR (Nanalysis Benchtop NMR spectrometer, 60 MHz, DMSO-*d_6_*, ppm): 13.73 (*s*, 1H, NH), 12.60 (*s*, 1H, OH), 9.34 (*s*, 1H, CH=N), 7.90–6.80 (*m*, 4H, aromatic H), 3.70–3.30 (*m*, 1H, cyc-butyl, CH), 2.55–1.75 (*m*, 6H, cyc-butyl, CH_2_).

Elemental analysis (Vario MACRO cube CHNS elemental analyzer): Found, %: C 64.31; H 5.64; N 23.79. C_13_H_14_N_4_O. Calculated, %: C, 64.45; H, 5.82; N, 23.13.

## Refinement

Crystal data, data collection and structure refinement details are summarized in Table 3 ▸. The O-bound H atom was located in a difference-Fourier map and refined with O—H = 0.82 Å, and with *U*_iso_(H) = 1.5*U*_eq_(O). The N-bound H atom was located in a difference-Fourier map. Its parameters were adjusted to give N—H = 0.86 Å and it was then refined as riding with *U*_iso_(H) =1.2*U*_eq_(N). The C-bound H atoms were positioned geometrically and refined using a riding model with C—H = 0.93 Å and *U*_iso_(H) = 1.2*U*_eq_(C) for aromatic and other H atoms, and with C—H = 0.97 Å and *U*_iso_(H) = 1.5*U*_eq_(C) for methyl­ene H atoms. The crystal studied was refined as a two-component inversion twin.

## Supplementary Material

Crystal structure: contains datablock(s) I. DOI: 10.1107/S2056989021011658/zv2010sup1.cif

Structure factors: contains datablock(s) I. DOI: 10.1107/S2056989021011658/zv2010Isup2.hkl

Supporting information file. DOI: 10.1107/S2056989021011658/zv2010Isup3.cml

CCDC reference: 2120120

Additional supporting information:  crystallographic information; 3D view; checkCIF report

## Figures and Tables

**Table 1:** Selected bond lengths (Å)

N11—N10	1.361 (5)	O1—C1	1.355 (6)
N6—N7	1.349 (5)	N3—N2	1.349 (6)
N5—C20	1.287 (5)	N1—C7	1.276 (6)
N9—C33	1.287 (6)	O3—C27	1.351 (6)
O2—C14	1.349 (5)		

**Table 2:** Hydrogen-bond geometry (Å, °)

*D*—H⋯*A*	*D*—H	H⋯*A*	*D*⋯*A*	*D*—H⋯*A*
N7—H7*D*⋯N2	0.86	1.98	2.836 (5)	174
N11—H11⋯N6	0.86	1.99	2.817 (5)	161
O1—H1⋯N1	0.82	1.89	2.615 (5)	146
O2—H2⋯N5	0.82	1.90	2.619 (4)	146
O3—H3⋯N9	0.82	1.87	2.588 (5)	146
C10—H10⋯O3^i^	0.98	2.52	3.411 (6)	151
N3—H3*D*⋯N10^ii^	0.86	2.07	2.874 (5)	155

Symmetry codes: (i) x-2, y, z; (ii) x-1, y, z.

**Table 3:** Experimental details

Crystal data	
Chemical formula	C_13_H_14_N_4_O
*M*_r_	242.28
Crystal system, space group	Monoclinic, *P*2_1_
Temperature (K)	296
*a*, *b*, *c* (Å)	5.2717 (3), 24.9066 (14), 14.8628 (7)
β (°)	96.214 (4)
*V* (Å^3^)	1940.02 (18)
*Z*	6
Radiation type	Mo *K*α
μ (mm^−1^)	0.08
Crystal size (mm)	0.76 × 0.52 × 0.30

Data collection	
Diffractometer	Stoe IPDS 2
Absorption correction	Integration (*X-RED32*; Stoe & Cie, 2002 ▸)
*T*_min_, *T*_max_	0.938, 0.980
No. of measured, independent and observed [*I* > 2σ(*I*)] reflections	15228, 8333, 5374
*R*_int_	0.096
(sin θ/λ)_max_ (Å^−1^)	0.637

Refinement	
*R*[*F* ^2^ > 2σ(*F* ^2^)], *wR*(*F* ^2^), *S*	0.058, 0.153, 0.94
No. of reflections	8333
No. of parameters	493
No. of restraints	1
H-atom treatment	H atoms treated by a mixture of independent and constrained refinement
Δρ_max_, Δρ_min_ (e Å^−3^)	0.16, −0.19
Absolute structure	Refined as an inversion twin
Absolute structure parameter	−1 (2)

Computer programs: *X-AREA* and *X-RED* (Stoe & Cie, 2002 ▸), *SHELXT2017/1* (Sheldrick, 2015*a* ▸), *SHELXL2017/1* (Sheldrick, 2015*b* ▸), *PLATON* (Spek, 2020 ▸) and *WinGX* (Farrugia, 2012 ▸).

## supplementary crystallographic information

### Crystal data

**Table:**

C_13_H_14_N_4_O	*F*(000) = 768
*M**_r_* = 242.28	*D*_x_ = 1.244 Mg m^−^^3^
Monoclinic, *P*2_1_	Mo *K*α radiation, λ = 0.71073 Å
*a* = 5.2717 (3) Å	Cell parameters from 17085 reflections
*b* = 24.9066 (14) Å	θ = 1.4–27.4°
*c* = 14.8628 (7) Å	µ = 0.08 mm^−^^1^
β = 96.214 (4)°	*T* = 296 K
*V* = 1940.02 (18) Å^3^	Stick, yellow
*Z* = 6	0.76 × 0.52 × 0.30 mm

### Data collection

**Table:**

Stoe IPDS 2 diffractometer	8333 independent reflections
Radiation source: sealed X-ray tube, 12 x 0.4 mm long-fine focus	5374 reflections with *I* > 2σ(*I*)
Detector resolution: 6.67 pixels mm^-1^	*R*_int_ = 0.096
rotation method scans	θ_max_ = 26.9°, θ_min_ = 1.4°
Absorption correction: integration (X-RED32; Stoe & Cie, 2002)	*h* = −6→6
*T*_min_ = 0.938, *T*_max_ = 0.980	*k* = −31→31
15228 measured reflections	*l* = −18→18

### Refinement

**Table:**

Refinement on *F*^2^	Hydrogen site location: inferred from neighbouring sites
Least-squares matrix: full	H atoms treated by a mixture of independent and constrained refinement
*R*[*F*^2^ > 2σ(*F*^2^)] = 0.058	*w* = 1/[σ^2^(*F*_o_^2^) + (0.084*P*)^2^] where *P* = (*F*_o_^2^ + 2*F*_c_^2^)/3
*wR*(*F*^2^) = 0.153	(Δ/σ)_max_ < 0.001
*S* = 0.94	Δρ_max_ = 0.16 e Å^−^^3^
8333 reflections	Δρ_min_ = −0.19 e Å^−^^3^
493 parameters	Absolute structure: Refined as an inversion twin
1 restraint	Absolute structure parameter: −1 (2)

### Special details

**Table:**

Geometry. All esds (except the esd in the dihedral angle between two l.s. planes) are estimated using the full covariance matrix. The cell esds are taken into account individually in the estimation of esds in distances, angles and torsion angles; correlations between esds in cell parameters are only used when they are defined by crystal symmetry. An approximate (isotropic) treatment of cell esds is used for estimating esds involving l.s. planes.
Refinement. Refined as a two-component inversion twin.

### Fractional atomic coordinates and isotropic or equivalent isotropic displacement parameters (Å^2^)

**Table:**

	*x*	*y*	*z*	*U*_iso_*/*U*_eq_	
N11	−0.7970 (7)	−0.44524 (14)	−0.4433 (2)	0.0699 (9)	
H11	−0.897406	−0.472528	−0.444919	0.084*	
N6	−1.0215 (7)	−0.54788 (13)	−0.4431 (2)	0.0652 (9)	
N5	−0.8325 (6)	−0.56917 (14)	−0.5739 (2)	0.0616 (8)	
N9	−0.3235 (7)	−0.36402 (14)	−0.3466 (2)	0.0699 (9)	
N12	−0.6141 (7)	−0.37213 (14)	−0.4855 (2)	0.0698 (9)	
O2	−0.4440 (6)	−0.51862 (14)	−0.6305 (2)	0.0792 (8)	
H2	−0.548954	−0.525821	−0.595446	0.119*	
N10	−0.6221 (8)	−0.43214 (14)	−0.3725 (2)	0.0709 (9)	
N7	−1.1902 (7)	−0.57344 (14)	−0.3961 (2)	0.0667 (9)	
H7D	−1.247216	−0.560968	−0.348178	0.088 (16)*	
O1	−0.7626 (7)	−0.65935 (15)	−0.2019 (2)	0.0900 (10)	
H1	−0.871881	−0.635799	−0.204508	0.135*	
N8	−1.1368 (7)	−0.62721 (14)	−0.5071 (2)	0.0645 (8)	
N3	−1.5053 (9)	−0.49514 (16)	−0.2114 (2)	0.0809 (11)	
H3D	−1.563359	−0.470387	−0.248339	0.077 (14)*	
N1	−1.1418 (7)	−0.60443 (14)	−0.1485 (2)	0.0673 (9)	
C21	−0.9973 (8)	−0.58229 (16)	−0.5094 (3)	0.0597 (9)	
N4	−1.4393 (8)	−0.54232 (16)	−0.0881 (2)	0.0779 (10)	
N2	−1.3418 (8)	−0.53404 (15)	−0.2310 (2)	0.0756 (10)	
C20	−0.8486 (8)	−0.59720 (17)	−0.6470 (3)	0.0615 (9)	
H20	−0.967147	−0.624910	−0.654904	0.074*	
C35	−0.7894 (9)	−0.40930 (16)	−0.5098 (3)	0.0654 (10)	
C34	−0.5166 (9)	−0.38873 (16)	−0.4011 (3)	0.0630 (10)	
O3	−0.0219 (9)	−0.35762 (17)	−0.1976 (3)	0.1155 (15)	
H3	−0.124553	−0.371866	−0.235635	0.173*	
C8	−1.3077 (9)	−0.56122 (17)	−0.1547 (3)	0.0673 (10)	
C7	−1.1444 (9)	−0.63770 (19)	−0.0833 (3)	0.0709 (11)	
H7	−1.257747	−0.631869	−0.040517	0.085*	
C32	−0.0082 (8)	−0.29555 (16)	−0.3204 (3)	0.0637 (10)	
C22	−1.2564 (8)	−0.62051 (16)	−0.4337 (3)	0.0633 (10)	
C33	−0.2150 (9)	−0.32178 (18)	−0.3745 (3)	0.0674 (11)	
H33	−0.271873	−0.307826	−0.431097	0.081*	
C19	−0.6849 (8)	−0.58627 (17)	−0.7172 (3)	0.0607 (9)	
C31	0.1081 (9)	−0.2502 (2)	−0.3523 (3)	0.0764 (12)	
H31	0.048601	−0.236737	−0.409058	0.092*	
C23	−1.4205 (9)	−0.66013 (18)	−0.3935 (3)	0.0721 (12)	
H23	−1.533394	−0.642112	−0.354781	0.087*	
C1	−0.7985 (8)	−0.69374 (19)	−0.1338 (3)	0.0713 (11)	
C6	−0.9800 (8)	−0.68365 (18)	−0.0732 (3)	0.0646 (10)	
C9	−1.5652 (11)	−0.5004 (2)	−0.1259 (3)	0.0799 (13)	
C15	−0.3418 (9)	−0.5393 (2)	−0.7774 (3)	0.0825 (13)	
H15	−0.211309	−0.513991	−0.770918	0.099*	
C14	−0.4932 (7)	−0.54795 (18)	−0.7067 (3)	0.0634 (10)	
C16	−0.3863 (11)	−0.5680 (3)	−0.8555 (3)	0.0887 (15)	
H16	−0.285384	−0.561698	−0.901974	0.106*	
C30	0.3059 (10)	−0.2248 (2)	−0.3032 (4)	0.0835 (13)	
H30	0.377953	−0.194138	−0.325682	0.100*	
C24	−1.2859 (11)	−0.7076 (2)	−0.3438 (4)	0.0945 (16)	
H24A	−1.275498	−0.704915	−0.278389	0.113*	
H24B	−1.120686	−0.715851	−0.363368	0.113*	
C5	−1.0037 (10)	−0.7208 (2)	−0.0045 (3)	0.0838 (13)	
H5	−1.123504	−0.714929	0.036028	0.101*	
C27	0.0858 (9)	−0.3150 (2)	−0.2347 (3)	0.0773 (13)	
C36	−0.9562 (9)	−0.41109 (19)	−0.5962 (3)	0.0741 (11)	
H36	−1.079123	−0.440553	−0.595341	0.089*	
C18	−0.7251 (9)	−0.6154 (2)	−0.7978 (3)	0.0758 (12)	
H18	−0.852386	−0.641378	−0.805055	0.091*	
C2	−0.6502 (10)	−0.7394 (2)	−0.1235 (4)	0.0910 (15)	
H2A	−0.529395	−0.745790	−0.163398	0.109*	
C4	−0.8513 (11)	−0.7665 (2)	0.0046 (4)	0.0957 (16)	
H4	−0.867080	−0.790781	0.051120	0.115*	
C26	−1.5682 (9)	−0.7009 (2)	−0.4542 (4)	0.0835 (14)	
H26A	−1.749634	−0.693456	−0.464529	0.100*	
H26B	−1.497645	−0.706884	−0.510986	0.100*	
C3	−0.6767 (12)	−0.7752 (3)	−0.0564 (4)	0.0997 (17)	
H3A	−0.575938	−0.805905	−0.051536	0.120*	
C10	−1.7219 (14)	−0.4618 (2)	−0.0811 (4)	0.106 (2)	
H10	−1.841507	−0.443208	−0.125701	0.127*	
C28	0.2882 (11)	−0.2889 (3)	−0.1857 (4)	0.0950 (16)	
H28	0.350184	−0.301504	−0.128671	0.114*	
C17	−0.5736 (11)	−0.6054 (3)	−0.8673 (3)	0.0888 (15)	
H17	−0.600936	−0.624342	−0.921459	0.107*	
C29	0.3970 (10)	−0.2450 (2)	−0.2204 (4)	0.0878 (14)	
H29	0.535293	−0.228508	−0.187238	0.105*	
C39	−1.0923 (12)	−0.3597 (3)	−0.6292 (4)	0.0994 (17)	
H39A	−1.271579	−0.358844	−0.619836	0.119*	
H39B	−1.006271	−0.327158	−0.606644	0.119*	
C25	−1.5013 (11)	−0.7440 (2)	−0.3854 (4)	0.0948 (16)	
H25A	−1.442921	−0.777109	−0.410537	0.114*	
H25B	−1.632828	−0.750774	−0.345929	0.114*	
C37	−0.8362 (12)	−0.4113 (3)	−0.6856 (3)	0.1036 (18)	
H37A	−0.667032	−0.395503	−0.681379	0.124*	
H37B	−0.839005	−0.446067	−0.715000	0.124*	
C38	−1.0446 (15)	−0.3741 (3)	−0.7240 (4)	0.121 (2)	
H38A	−1.186802	−0.392224	−0.758146	0.145*	
H38B	−0.984828	−0.344306	−0.758075	0.145*	
C11	−1.5830 (19)	−0.4224 (4)	−0.0169 (8)	0.167 (4)	
H11A	−1.414523	−0.434387	0.007721	0.200*	
H11B	−1.576586	−0.386355	−0.041158	0.200*	
C12	−1.776 (2)	−0.4295 (5)	0.0446 (6)	0.177 (4)	
H12A	−1.907206	−0.401994	0.038835	0.212*	
H12B	−1.706473	−0.433875	0.107314	0.212*	
C13	−1.8566 (14)	−0.4810 (4)	−0.0013 (5)	0.125 (2)	
H13A	−2.039826	−0.484886	−0.014726	0.150*	
H13B	−1.781036	−0.512679	0.028581	0.150*	

### Atomic displacement parameters (Å^2^)

**Table:**

	*U*^11^	*U*^22^	*U*^33^	*U*^12^	*U*^13^	*U*^23^
N11	0.097 (2)	0.0553 (18)	0.0566 (19)	−0.0203 (18)	0.0055 (17)	−0.0031 (15)
N6	0.092 (2)	0.0555 (18)	0.0513 (17)	−0.0169 (17)	0.0211 (16)	−0.0036 (15)
N5	0.0716 (18)	0.0616 (18)	0.0541 (17)	−0.0049 (16)	0.0186 (14)	−0.0007 (15)
N9	0.096 (3)	0.059 (2)	0.0516 (17)	−0.0067 (19)	−0.0049 (17)	0.0045 (15)
N12	0.093 (2)	0.060 (2)	0.0533 (18)	−0.0144 (18)	−0.0069 (16)	0.0124 (15)
O2	0.088 (2)	0.086 (2)	0.0657 (18)	−0.0154 (17)	0.0176 (14)	−0.0036 (16)
N10	0.105 (3)	0.0541 (19)	0.0528 (18)	−0.0139 (18)	0.0040 (18)	0.0043 (15)
N7	0.097 (2)	0.0576 (19)	0.0495 (17)	−0.0128 (18)	0.0262 (17)	−0.0061 (15)
O1	0.097 (2)	0.092 (2)	0.086 (2)	0.0105 (19)	0.0338 (18)	0.0083 (19)
N8	0.078 (2)	0.0558 (19)	0.0634 (19)	−0.0051 (17)	0.0253 (16)	−0.0067 (15)
N3	0.133 (3)	0.063 (2)	0.0467 (18)	0.016 (2)	0.0101 (19)	0.0084 (16)
N1	0.089 (2)	0.063 (2)	0.0517 (18)	0.0084 (18)	0.0155 (16)	0.0017 (15)
C21	0.073 (2)	0.055 (2)	0.053 (2)	−0.0082 (19)	0.0154 (17)	−0.0015 (17)
N4	0.120 (3)	0.073 (2)	0.0427 (16)	0.028 (2)	0.0158 (17)	0.0011 (16)
N2	0.121 (3)	0.061 (2)	0.0473 (18)	0.007 (2)	0.0200 (18)	0.0027 (15)
C20	0.071 (2)	0.058 (2)	0.058 (2)	−0.0010 (19)	0.0162 (18)	−0.0006 (17)
C35	0.085 (3)	0.053 (2)	0.057 (2)	−0.010 (2)	−0.0011 (19)	0.0053 (18)
C34	0.088 (3)	0.052 (2)	0.047 (2)	−0.008 (2)	−0.0001 (18)	0.0050 (16)
O3	0.158 (4)	0.100 (3)	0.077 (2)	−0.045 (3)	−0.040 (2)	0.035 (2)
C8	0.098 (3)	0.062 (2)	0.045 (2)	0.007 (2)	0.0171 (19)	−0.0001 (17)
C7	0.087 (3)	0.076 (3)	0.051 (2)	0.008 (2)	0.0143 (19)	−0.004 (2)
C32	0.078 (2)	0.058 (2)	0.053 (2)	0.000 (2)	−0.0030 (18)	0.0000 (18)
C22	0.078 (2)	0.055 (2)	0.060 (2)	−0.008 (2)	0.0214 (19)	−0.0068 (18)
C33	0.089 (3)	0.060 (2)	0.050 (2)	−0.002 (2)	−0.0051 (19)	0.0058 (18)
C19	0.066 (2)	0.066 (2)	0.052 (2)	0.0133 (19)	0.0129 (17)	0.0042 (17)
C31	0.089 (3)	0.071 (3)	0.065 (3)	−0.010 (2)	−0.008 (2)	0.009 (2)
C23	0.083 (3)	0.065 (2)	0.075 (3)	−0.015 (2)	0.035 (2)	−0.010 (2)
C1	0.069 (2)	0.074 (3)	0.070 (3)	0.000 (2)	0.000 (2)	−0.006 (2)
C6	0.072 (2)	0.064 (2)	0.056 (2)	0.004 (2)	−0.0003 (18)	−0.0023 (18)
C9	0.121 (4)	0.075 (3)	0.044 (2)	0.019 (3)	0.008 (2)	−0.003 (2)
C15	0.078 (3)	0.098 (3)	0.074 (3)	−0.004 (3)	0.025 (2)	0.018 (3)
C14	0.065 (2)	0.074 (2)	0.052 (2)	0.007 (2)	0.0095 (17)	0.0100 (18)
C16	0.097 (3)	0.113 (4)	0.060 (3)	0.019 (3)	0.028 (2)	0.013 (3)
C30	0.088 (3)	0.075 (3)	0.085 (3)	−0.011 (3)	−0.004 (2)	0.004 (2)
C24	0.097 (3)	0.100 (4)	0.084 (3)	−0.030 (3)	−0.002 (3)	0.025 (3)
C5	0.096 (3)	0.089 (3)	0.065 (3)	0.011 (3)	0.001 (2)	0.014 (2)
C27	0.098 (3)	0.071 (3)	0.059 (3)	−0.008 (2)	−0.011 (2)	0.008 (2)
C36	0.084 (3)	0.068 (3)	0.068 (3)	−0.017 (2)	−0.005 (2)	0.003 (2)
C18	0.084 (3)	0.086 (3)	0.059 (2)	0.007 (2)	0.017 (2)	−0.007 (2)
C2	0.083 (3)	0.099 (4)	0.091 (4)	0.020 (3)	0.006 (3)	0.001 (3)
C4	0.114 (4)	0.086 (3)	0.084 (4)	0.013 (3)	−0.008 (3)	0.023 (3)
C26	0.075 (3)	0.084 (3)	0.093 (3)	−0.019 (2)	0.013 (2)	−0.003 (3)
C3	0.102 (4)	0.093 (4)	0.100 (4)	0.035 (3)	−0.008 (3)	0.000 (3)
C10	0.161 (5)	0.091 (4)	0.064 (3)	0.059 (4)	0.001 (3)	0.002 (3)
C28	0.114 (4)	0.099 (4)	0.064 (3)	−0.013 (3)	−0.027 (3)	0.006 (3)
C17	0.097 (3)	0.117 (4)	0.055 (2)	0.015 (3)	0.019 (2)	−0.004 (3)
C29	0.089 (3)	0.088 (3)	0.082 (3)	−0.016 (3)	−0.011 (3)	−0.009 (3)
C39	0.099 (4)	0.100 (4)	0.093 (4)	0.017 (3)	−0.016 (3)	−0.006 (3)
C25	0.103 (4)	0.073 (3)	0.110 (4)	−0.020 (3)	0.021 (3)	−0.009 (3)
C37	0.113 (4)	0.136 (5)	0.058 (3)	0.020 (4)	−0.006 (3)	−0.006 (3)
C38	0.142 (5)	0.139 (6)	0.077 (4)	0.015 (5)	−0.010 (3)	0.022 (4)
C11	0.174 (7)	0.119 (6)	0.216 (10)	−0.016 (6)	0.064 (7)	−0.104 (7)
C12	0.224 (10)	0.199 (10)	0.109 (6)	0.031 (9)	0.022 (6)	−0.063 (7)
C13	0.120 (5)	0.137 (6)	0.126 (6)	0.026 (4)	0.043 (4)	−0.021 (5)

### Geometric parameters (Å, º)

**Table:**

N11—C35	1.338 (5)	C15—C16	1.361 (8)
N11—N10	1.361 (5)	C15—C14	1.403 (6)
N11—H11	0.8600	C15—H15	0.9300
N6—C21	1.322 (5)	C16—C17	1.356 (8)
N6—N7	1.349 (5)	C16—H16	0.9300
N5—C20	1.287 (5)	C30—C29	1.369 (7)
N5—C21	1.400 (5)	C30—H30	0.9300
N9—C33	1.287 (6)	C24—C25	1.530 (8)
N9—C34	1.376 (5)	C24—H24A	0.9700
N12—C35	1.330 (5)	C24—H24B	0.9700
N12—C34	1.368 (5)	C5—C4	1.391 (8)
O2—C14	1.349 (5)	C5—H5	0.9300
O2—H2	0.8200	C27—C28	1.386 (7)
N10—C34	1.308 (5)	C36—C39	1.523 (7)
N7—C22	1.330 (5)	C36—C37	1.533 (8)
N7—H7D	0.8600	C36—H36	0.9800
O1—C1	1.355 (6)	C18—C17	1.395 (7)
O1—H1	0.8200	C18—H18	0.9300
N8—C22	1.328 (5)	C2—C3	1.355 (9)
N8—C21	1.341 (5)	C2—H2A	0.9300
N3—C9	1.347 (6)	C4—C3	1.377 (8)
N3—N2	1.349 (6)	C4—H4	0.9300
N3—H3D	0.8600	C26—C25	1.498 (8)
N1—C7	1.276 (6)	C26—H26A	0.9700
N1—C8	1.383 (6)	C26—H26B	0.9700
N4—C9	1.329 (6)	C3—H3A	0.9300
N4—C8	1.353 (5)	C10—C11	1.503 (10)
N2—C8	1.316 (5)	C10—C13	1.524 (10)
C20—C19	1.451 (5)	C10—H10	0.9800
C20—H20	0.9300	C28—C29	1.361 (8)
C35—C36	1.476 (6)	C28—H28	0.9300
O3—C27	1.351 (6)	C17—H17	0.9300
O3—H3	0.8200	C29—H29	0.9300
C7—C6	1.434 (6)	C39—C38	1.502 (9)
C7—H7	0.9300	C39—H39A	0.9700
C32—C31	1.393 (6)	C39—H39B	0.9700
C32—C27	1.402 (6)	C25—H25A	0.9700
C32—C33	1.439 (6)	C25—H25B	0.9700
C22—C23	1.481 (6)	C37—C38	1.501 (9)
C33—H33	0.9300	C37—H37A	0.9700
C19—C14	1.386 (6)	C37—H37B	0.9700
C19—C18	1.396 (6)	C38—H38A	0.9700
C31—C30	1.362 (7)	C38—H38B	0.9700
C31—H31	0.9300	C11—C12	1.450 (13)
C23—C26	1.517 (7)	C11—H11A	0.9700
C23—C24	1.527 (8)	C11—H11B	0.9700
C23—H23	0.9800	C12—C13	1.493 (13)
C1—C2	1.379 (7)	C12—H12A	0.9700
C1—C6	1.406 (6)	C12—H12B	0.9700
C6—C5	1.394 (7)	C13—H13A	0.9700
C9—C10	1.472 (7)	C13—H13B	0.9700
			
C35—N11—N10	109.8 (3)	C4—C5—C6	121.1 (5)
C35—N11—H11	125.1	C4—C5—H5	119.4
N10—N11—H11	125.1	C6—C5—H5	119.4
C21—N6—N7	101.7 (3)	O3—C27—C28	119.2 (4)
C20—N5—C21	117.5 (3)	O3—C27—C32	121.3 (4)
C33—N9—C34	120.1 (3)	C28—C27—C32	119.5 (4)
C35—N12—C34	102.7 (3)	C35—C36—C39	118.1 (4)
C14—O2—H2	109.5	C35—C36—C37	119.4 (4)
C34—N10—N11	103.0 (3)	C39—C36—C37	87.0 (4)
C22—N7—N6	110.8 (3)	C35—C36—H36	110.1
C22—N7—H7D	124.6	C39—C36—H36	110.1
N6—N7—H7D	124.6	C37—C36—H36	110.1
C1—O1—H1	109.5	C17—C18—C19	119.8 (5)
C22—N8—C21	103.1 (3)	C17—C18—H18	120.1
C9—N3—N2	110.8 (4)	C19—C18—H18	120.1
C9—N3—H3D	124.6	C3—C2—C1	121.4 (5)
N2—N3—H3D	124.6	C3—C2—H2A	119.3
C7—N1—C8	119.8 (4)	C1—C2—H2A	119.3
N6—C21—N8	115.1 (3)	C3—C4—C5	119.0 (5)
N6—C21—N5	118.4 (3)	C3—C4—H4	120.5
N8—C21—N5	126.5 (3)	C5—C4—H4	120.5
C9—N4—C8	103.7 (4)	C25—C26—C23	90.3 (4)
C8—N2—N3	102.4 (3)	C25—C26—H26A	113.6
N5—C20—C19	121.1 (4)	C23—C26—H26A	113.6
N5—C20—H20	119.5	C25—C26—H26B	113.6
C19—C20—H20	119.5	C23—C26—H26B	113.6
N12—C35—N11	109.9 (3)	H26A—C26—H26B	110.9
N12—C35—C36	126.0 (4)	C2—C3—C4	120.7 (5)
N11—C35—C36	124.1 (4)	C2—C3—H3A	119.7
N10—C34—N12	114.6 (4)	C4—C3—H3A	119.7
N10—C34—N9	119.5 (4)	C9—C10—C11	117.0 (6)
N12—C34—N9	125.9 (4)	C9—C10—C13	118.6 (5)
C27—O3—H3	109.5	C11—C10—C13	86.9 (6)
N2—C8—N4	114.7 (4)	C9—C10—H10	110.8
N2—C8—N1	118.9 (3)	C11—C10—H10	110.8
N4—C8—N1	126.4 (4)	C13—C10—H10	110.8
N1—C7—C6	122.8 (4)	C29—C28—C27	120.6 (5)
N1—C7—H7	118.6	C29—C28—H28	119.7
C6—C7—H7	118.6	C27—C28—H28	119.7
C31—C32—C27	117.6 (4)	C16—C17—C18	119.6 (5)
C31—C32—C33	120.9 (4)	C16—C17—H17	120.2
C27—C32—C33	121.5 (4)	C18—C17—H17	120.2
N8—C22—N7	109.3 (3)	C28—C29—C30	121.0 (5)
N8—C22—C23	126.8 (4)	C28—C29—H29	119.5
N7—C22—C23	123.7 (4)	C30—C29—H29	119.5
N9—C33—C32	121.9 (4)	C38—C39—C36	88.9 (5)
N9—C33—H33	119.1	C38—C39—H39A	113.8
C32—C33—H33	119.1	C36—C39—H39A	113.8
C14—C19—C18	119.7 (4)	C38—C39—H39B	113.8
C14—C19—C20	122.0 (4)	C36—C39—H39B	113.8
C18—C19—C20	118.2 (4)	H39A—C39—H39B	111.1
C30—C31—C32	122.3 (4)	C26—C25—C24	87.8 (4)
C30—C31—H31	118.8	C26—C25—H25A	114.0
C32—C31—H31	118.8	C24—C25—H25A	114.0
C22—C23—C26	119.4 (4)	C26—C25—H25B	114.0
C22—C23—C24	116.7 (4)	C24—C25—H25B	114.0
C26—C23—C24	87.2 (4)	H25A—C25—H25B	111.2
C22—C23—H23	110.5	C38—C37—C36	88.5 (5)
C26—C23—H23	110.5	C38—C37—H37A	113.9
C24—C23—H23	110.5	C36—C37—H37A	113.9
O1—C1—C2	118.8 (5)	C38—C37—H37B	113.9
O1—C1—C6	121.7 (4)	C36—C37—H37B	113.9
C2—C1—C6	119.6 (5)	H37A—C37—H37B	111.1
C5—C6—C1	118.2 (4)	C37—C38—C39	88.9 (4)
C5—C6—C7	120.4 (4)	C37—C38—H38A	113.8
C1—C6—C7	121.4 (4)	C39—C38—H38A	113.8
N4—C9—N3	108.5 (4)	C37—C38—H38B	113.8
N4—C9—C10	126.9 (4)	C39—C38—H38B	113.8
N3—C9—C10	124.3 (4)	H38A—C38—H38B	111.1
C16—C15—C14	119.9 (5)	C12—C11—C10	89.7 (7)
C16—C15—H15	120.1	C12—C11—H11A	113.7
C14—C15—H15	120.1	C10—C11—H11A	113.7
O2—C14—C19	122.7 (4)	C12—C11—H11B	113.7
O2—C14—C15	118.2 (4)	C10—C11—H11B	113.7
C19—C14—C15	119.2 (4)	H11A—C11—H11B	110.9
C17—C16—C15	121.9 (5)	C11—C12—C13	90.0 (6)
C17—C16—H16	119.1	C11—C12—H12A	113.6
C15—C16—H16	119.1	C13—C12—H12A	113.6
C31—C30—C29	119.0 (5)	C11—C12—H12B	113.6
C31—C30—H30	120.5	C13—C12—H12B	113.6
C29—C30—H30	120.5	H12A—C12—H12B	110.9
C23—C24—C25	88.7 (4)	C12—C13—C10	87.3 (7)
C23—C24—H24A	113.9	C12—C13—H13A	114.1
C25—C24—H24A	113.9	C10—C13—H13A	114.1
C23—C24—H24B	113.9	C12—C13—H13B	114.1
C25—C24—H24B	113.9	C10—C13—H13B	114.1
H24A—C24—H24B	111.1	H13A—C13—H13B	111.3
			
C35—N11—N10—C34	−0.4 (5)	C20—C19—C14—O2	0.6 (6)
C21—N6—N7—C22	0.6 (5)	C18—C19—C14—C15	−0.3 (6)
N7—N6—C21—N8	−0.2 (5)	C20—C19—C14—C15	−179.9 (4)
N7—N6—C21—N5	180.0 (4)	C16—C15—C14—O2	−179.7 (5)
C22—N8—C21—N6	−0.3 (5)	C16—C15—C14—C19	0.7 (7)
C22—N8—C21—N5	179.5 (4)	C14—C15—C16—C17	−0.4 (8)
C20—N5—C21—N6	−166.4 (4)	C32—C31—C30—C29	1.2 (8)
C20—N5—C21—N8	13.8 (6)	C22—C23—C24—C25	−139.8 (4)
C9—N3—N2—C8	−0.6 (5)	C26—C23—C24—C25	−18.1 (4)
C21—N5—C20—C19	−180.0 (4)	C1—C6—C5—C4	0.4 (7)
C34—N12—C35—N11	0.7 (5)	C7—C6—C5—C4	178.0 (5)
C34—N12—C35—C36	179.8 (5)	C31—C32—C27—O3	177.7 (5)
N10—N11—C35—N12	−0.2 (5)	C33—C32—C27—O3	−3.1 (8)
N10—N11—C35—C36	−179.3 (4)	C31—C32—C27—C28	−0.3 (7)
N11—N10—C34—N12	0.9 (5)	C33—C32—C27—C28	178.9 (5)
N11—N10—C34—N9	−179.0 (4)	N12—C35—C36—C39	−47.2 (7)
C35—N12—C34—N10	−1.1 (5)	N11—C35—C36—C39	131.7 (5)
C35—N12—C34—N9	178.9 (5)	N12—C35—C36—C37	56.2 (7)
C33—N9—C34—N10	178.8 (4)	N11—C35—C36—C37	−124.8 (5)
C33—N9—C34—N12	−1.1 (7)	C14—C19—C18—C17	−0.5 (7)
N3—N2—C8—N4	0.3 (5)	C20—C19—C18—C17	179.1 (4)
N3—N2—C8—N1	−178.3 (4)	O1—C1—C2—C3	179.8 (5)
C9—N4—C8—N2	0.2 (6)	C6—C1—C2—C3	0.5 (8)
C9—N4—C8—N1	178.6 (5)	C6—C5—C4—C3	−0.8 (9)
C7—N1—C8—N2	−165.7 (4)	C22—C23—C26—C25	137.8 (5)
C7—N1—C8—N4	15.9 (7)	C24—C23—C26—C25	18.5 (4)
C8—N1—C7—C6	179.3 (4)	C1—C2—C3—C4	−0.9 (9)
C21—N8—C22—N7	0.6 (5)	C5—C4—C3—C2	1.1 (9)
C21—N8—C22—C23	−174.3 (4)	N4—C9—C10—C11	72.4 (9)
N6—N7—C22—N8	−0.8 (5)	N3—C9—C10—C11	−100.3 (8)
N6—N7—C22—C23	174.3 (4)	N4—C9—C10—C13	−29.6 (10)
C34—N9—C33—C32	−178.4 (4)	N3—C9—C10—C13	157.6 (6)
C31—C32—C33—N9	179.4 (4)	O3—C27—C28—C29	−178.4 (6)
C27—C32—C33—N9	0.2 (7)	C32—C27—C28—C29	−0.5 (9)
N5—C20—C19—C14	4.4 (6)	C15—C16—C17—C18	−0.4 (8)
N5—C20—C19—C18	−175.2 (4)	C19—C18—C17—C16	0.9 (8)
C27—C32—C31—C30	−0.1 (7)	C27—C28—C29—C30	1.6 (9)
C33—C32—C31—C30	−179.3 (5)	C31—C30—C29—C28	−1.9 (9)
N8—C22—C23—C26	−29.5 (7)	C35—C36—C39—C38	141.3 (5)
N7—C22—C23—C26	156.2 (4)	C37—C36—C39—C38	19.3 (5)
N8—C22—C23—C24	73.2 (6)	C23—C26—C25—C24	−18.5 (4)
N7—C22—C23—C24	−101.0 (5)	C23—C24—C25—C26	18.4 (4)
O1—C1—C6—C5	−179.5 (4)	C35—C36—C37—C38	−140.0 (5)
C2—C1—C6—C5	−0.2 (7)	C39—C36—C37—C38	−19.3 (5)
O1—C1—C6—C7	3.0 (6)	C36—C37—C38—C39	19.6 (5)
C2—C1—C6—C7	−177.8 (4)	C36—C39—C38—C37	−19.7 (5)
N1—C7—C6—C5	−175.3 (5)	C9—C10—C11—C12	−139.3 (7)
N1—C7—C6—C1	2.2 (7)	C13—C10—C11—C12	−18.5 (8)
C8—N4—C9—N3	−0.6 (6)	C10—C11—C12—C13	18.9 (8)
C8—N4—C9—C10	−174.3 (6)	C11—C12—C13—C10	−18.7 (8)
N2—N3—C9—N4	0.8 (6)	C9—C10—C13—C12	137.3 (7)
N2—N3—C9—C10	174.7 (5)	C11—C10—C13—C12	18.0 (7)
C18—C19—C14—O2	−179.7 (4)		

### Hydrogen-bond geometry (Å, º)

**Table:**

*D*—H···*A*	*D*—H	H···*A*	*D*···*A*	*D*—H···*A*
N7—H7*D*···N2	0.86	1.98	2.836 (5)	174
N11—H11···N6	0.86	1.99	2.817 (5)	161
O1—H1···N1	0.82	1.89	2.615 (5)	146
O2—H2···N5	0.82	1.90	2.619 (4)	146
O3—H3···N9	0.82	1.87	2.588 (5)	146
C10—H10···O3^i^	0.98	2.52	3.411 (6)	151
N3—H3*D*···N10^ii^	0.86	2.07	2.874 (5)	155

Symmetry codes: (i) *x*−2, *y*, *z*; (ii) *x*−1, *y*, *z*.
